# Comparative Analysis of Production Performance and Physiological Responses in Snowy White Chickens Reared at Different Altitudes

**DOI:** 10.3390/life16060912

**Published:** 2026-05-28

**Authors:** Mingzhu Shan, Yang Liu, Tong Li, Yingjie Wang, Gang Shu, Liuting Wu, Xiaoling Zhao

**Affiliations:** 1 State Key Laboratory of Swine and Poultry Breeding Industry, College of Animal Science and Technology, Sichuan Agricultural University, Chengdu 611130, China; shanmz2001@gmail.com (M.S.); liu3045693128@163.com (Y.L.); litongheau@163.com (T.L.); wangyingjie@sicau.edu.cn (Y.W.); 2 Department of Basic Veterinary Medicine, Sichuan Agricultural University, Chengdu 611130, China; dyysg2005@sicau.edu.cn

**Keywords:** snowy white chicken, high altitude adaptation, body measurements, reproductive performance, physiological response

## Abstract

This study evaluated variations in phenotypic and physiological traits of Snowy White chickens reared under high-altitude conditions in Lhasa, China, at 3650 m and low-altitude conditions in Ya’an, China, at 600 m. Chickens reared at high altitude showed delayed sexual maturity and peak laying, as well as lower laying rate and hatchability. In contrast, egg weight at first laying was higher in chickens reared at high altitude. Organ index analysis showed that high-altitude chickens had a higher heart index but lower liver, stomach, and spleen indices than low-altitude chickens (*p* < 0.05). High-altitude chickens also had greater chest depth and chest circumference but shorter shank length and smaller shank circumference (*p* < 0.05). Multivariate analyses further indicated liver and spleen indices as major contributors to the separation between altitude groups. These results show that high-altitude rearing is associated with altered reproductive performance, organ development, and body conformation in Snowy White chickens. These findings may inform the evaluation, breeding, and management of layer chickens in plateau production systems.

## 1. Introduction

Plateau environments, characterized by hypoxia, low temperature, and intense ultraviolet radiation, impose substantial challenges on poultry growth, development, productive performance, and physiological homeostasis [1]. For decades, phenotypic adaptation in laying hens in high-altitude environments, alongside concurrent changes in their egg production and reproductive capabilities, has been a central focus of research within the fields of animal genetics and breeding [2,3]. Maintaining stable reproductive performance under extreme environmental conditions is important for sustainable poultry production and food security in plateau regions. The Snowy White Chicken, a distinct laying hen breed indigenous to China’s alpine regions, was developed through the hybridization of White Leghorn and Tibetan chickens. This breeding strategy has endowed the breed with the exceptional plateau adaptability of Tibetan chickens and the superior egg-laying performance of White Leghorns [4]. Therefore, this breed is relevant both for egg production in high-altitude regions and for investigating altitude-associated physiological adaptation in poultry. The development and utilization of this breed may also enrich plateau poultry genetic resources and support regional poultry production [3].

Previous studies have shown that high-altitude environments can impair reproductive performance of laying hens, leading to delayed age at first laying, reduced egg production rates, and a postponed peak in egg output [5,6,7]. Experimental evidence from hypoxic incubation studies has shown that mild hypoxia during embryogenesis can impair embryonic growth and alter hatchability and chick quality [8]. Studies in birds also indicate that high-altitude exposure places demands on the oxygen transport cascade, including pulmonary ventilation, oxygen diffusion, circulatory delivery, and tissue-level oxygen utilization [9]. Physiologically, plateau poultry often respond to hypoxic stress by increasing red blood cell counts and hemoglobin concentrations [10,11], alongside organ-level adaptations such as heart hypertrophy and lung structural optimization [12,13]. Mechanistically, key genes, particularly HIF-1α, and their associated regulatory networks are instrumental in mediating the hypoxic response [14,15].

Although previous studies have examined high-altitude adaptation in plateau animals from multiple perspectives, productive traits and physiological traits have often been evaluated separately. This makes it difficult to determine how multiple trait categories respond jointly to altitude within the same breed and under a relatively consistent genetic background. High-altitude poultry systems face challenges related to growth performance and physiological stress, highlighting the importance of nutritional, management, and breeding strategies [16]. Although high-altitude adaptation has been reported for several chicken populations, evidence remains limited regarding whether reproductive performance, body-size characteristics, and organ development undergo coordinated changes across different altitudinal conditions. Systematic phenotypic comparisons of this breed between high- and low-altitude environments remain scarce, leaving its comprehensive altitude-associated adaptation patterns insufficiently characterized.

In this study, we systematically compared the reproductive traits, body conformation traits, and organ indices of Snowy White chickens reared under high- and low-altitude environments. By integrating univariate and multivariate statistical approaches, we aimed to characterize altitude-associated differences in production performance, physiological traits, and body conformation traits under two field rearing environments. These findings provide a descriptive basis for understanding phenotypic variation in plateau poultry production systems.

## 2. Materials and Methods

### 2.1. Experimental Locations and Animals

**Figure 1:**
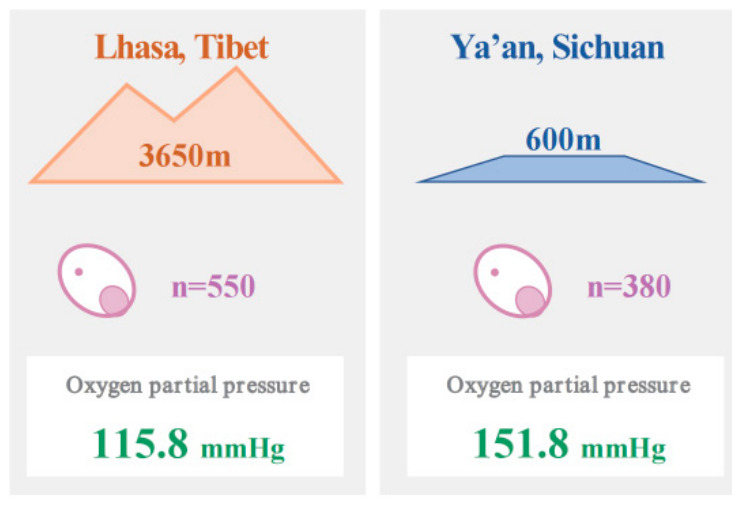
Rearing environments of Snowy White chickens.

In this study, Snowy White chickens reared under high- and low-altitude conditions were used as the experimental animals. The birds at both locations were Snowy White chickens derived from the same breeding batch and provided by the Institute of Animal Husbandry, Lhasa, Tibet, China. They were from the same generation and were managed according to the same breed-specific technical specifications. The specific rearing locations and environmental conditions are shown in Figure 1. The low-altitude group (*n* = 380) was maintained at the Poultry Breeding Unit, Teaching and Research Facility, Ya’an Campus, Sichuan Agricultural University, China. The high-altitude group (*n* = 550) was housed at the Institute of Animal Husbandry, Lhasa, Tibet, China. Group sizes were determined by the flock management capacity of the respective facilities and by the need to accommodate potential attrition during the long-term experiment. Embryo incubation was performed as described by Peng et al. [17].

### 2.2. Husbandry and Management of Experimental Animals

Routine husbandry practices and feeding protocols at both altitudinal sites followed the Technical Specifications for Lhasa White Chicken (Breed Group) Breeding (DB54/T0036-2021) [18]. A unified photoperiod regimen was applied in both groups: chicks received 23 h of light daily during days 1–2, which was subsequently reduced to 18–20 h after the first week. Thereafter, light duration was decreased by 0.5 h per week until reaching 14 h at 6 weeks of age. Light intensity was regulated at $4\;{W/m}^{3}$ during weeks 1–2, $2.5\;{W/m}^{3}$ lux during weeks 3–4, and $1.7\;{W/m}^{3}$ during weeks 5–6. From 20 weeks of age, artificial lighting was incrementally increased by 0.5–1 h per week until a constant 16-h photoperiod (combined artificial and natural light) was established at 25 weeks. The housing system and stocking density were standardized as far as possible between the two altitudinal sites throughout data collection. Birds in both groups were maintained in environmentally managed poultry houses under a cage-based system. During the brooding period (weeks 1–6; Supplementary Table S1), chickens were housed in brooding cages at a stocking density of 20 chickens/m^2^. After brooding, chickens in both groups were transferred to individual layer cages, with one bird per cage. Each cage measured 30 cm × 35 cm × 40 cm, corresponding to 1050 cm^2^ of floor space per chicken. This housing arrangement was maintained throughout the laying-performance recording period and until organ index and body conformation measurements were performed at 29 weeks of age. The cage-rearing system, feeding equipment, lighting program, biosecurity procedures, vaccination schedule, and stocking density were kept consistent between the two sites according to DB54/T0036-2021. Ambient temperature and humidity were maintained within the prescribed ranges for each growth stage in both environments (Supplementary Table S2). Throughout the experiment, all chickens received the same corn-soybean meal-based diet (New Hope Group Co., Ltd., Chengdu, China); basal diet composition and nutrient levels are provided in Supplementary Table S3. Because the experiment was conducted at two geographically distinct field facilities, minor site-specific microenvironmental differences could not be completely excluded. Therefore, the results were interpreted as altitude-associated responses under standardized cage-rearing, stocking-density, and management conditions. Standardized biosecurity, disinfection, and vaccination protocols were implemented at the specified ages (Supplementary Table S4).

### 2.3. Data Acquisition and Performance Metrics

#### 2.3.1. Measurement of Production Performance

##### Egg Production and Reproductive Metrics

Eggs were collected daily at a fixed time (11:00 AM), and total egg counts were recorded. The evaluated metrics were defined as follows:

- Age at first laying (days): the age at which the flock reached a cumulative laying rate of 50%.
- Egg weight at first laying (g): the average weight of eggs randomly sampled on the day the flock attained a 50% laying rate.
- Peak laying rate (%): the maximum daily laying rate achieved by the flock during the experimental cycle. The daily laying rate was calculated as follows:
  (1) $$Peak\;laying\;rate\;(\%)=\frac{Daily\;egg\;production}{Number\;of\;laying\;hens}\times 100$$
- Fertility rate (%): the proportion of fertile eggs relative to the total number of eggs set for incubation, calculated as follows:
  (2) $$Fertility\;rate\;(\%)=\frac{Number\;of\;fertilized\;eggs}{Number\;of\;eggs}\times 100$$

##### Assessment of Reproductive Performance Parameters

Before the collection of hatching eggs, sexually mature males from the same Snowy White chicken population were managed under the same site-specific husbandry conditions as the corresponding females. The male-to-female ratio was maintained at 1:9. To evaluate the fertility and hatchability of the eggs, artificial insemination was performed. The protocol involved an initial phase of insemination for three consecutive days, followed by a maintenance frequency of once every two days. Semen was collected and immediately inseminated into the hens within 2 min to ensure optimal sperm viability. All insemination procedures were conducted consistently across both the high- and low-altitude groups to eliminate methodology-induced variations.

Reproductive performance was evaluated based on the following parameters. For hatching-related traits, eggs from each altitude group were divided into three independent incubation replicates. Each replicate was managed and recorded separately, and fertility rate, hatchability of fertile eggs, hatchability of set eggs, healthy chick rate, and embryonic mortality rate were calculated independently for each replicate. Therefore, the incubation replicate, rather than the individual egg, was used as the experimental unit for statistical analysis of hatching traits. Values in Table 1 and Table 2 are presented as mean ± SD based on three independent incubation replicates per altitude group.

- Number of eggs set for incubation: The total number of hatching eggs placed in the incubator during the experimental trial.
- Hatchability of fertile eggs (%): The proportion of hatched chicks relative to the number of fertile eggs, calculated as follows:

(3) $$Hatchability\;of\;fertile\;eggs\;(\%)=\frac{Number\;of\;chicks\;hatched}{Number\;of\;fertilized\;eggs}\times 100$$

- Hatchability of set eggs (%): The proportion of chicks hatched relative to the total number of eggs set for incubation, calculated as follows:

(4) $$Hatchability\;of\;set\;eggs\;(\%)=\frac{Number\;of\;chicks\;hatched}{Number\;of\;incubated\;eggs}\times 100$$

- Healthy chick rate (%): The proportion of viable and healthy chicks among the total number of hatched chicks.

(5) $$Healthy\;chick\;rate\;(\%)=\frac{Number\;of\;healthy\;chicks}{Number\;of\;chicks\;hatched}\times 100$$

- Embryonic mortality rate (%): The proportion of embryos that died during incubation relative to the total number of eggs set for incubation, calculated as follows:

(6) $$Embryonic\;mortality\;rate\;(\%)=\frac{Number\;of\;dead\;embryos}{Number\;of\;incubated\;eggs}\times 100$$

- Statistical unit of analysis: the unit of analysis differed according to trait type. For production traits derived from flock-level records, including age at first laying and timing of peak egg production, the flock was the observational unit. For egg weight at first laying, each randomly sampled intact egg was used as the measurement unit. For organ indices and body conformation traits, each sampled hen was used as the individual experimental unit. For hatching traits, each incubation batch or replicate record was used as the unit of analysis, as appropriate. Statistical comparisons were performed only when replication was compatible with the corresponding statistical test.

#### 2.3.2. Physiological Parameter Determination

##### Organ Index Measurement

At 29 weeks of age, corresponding to the age at first laying in the high-altitude group, 12 healthy hens were selected from each altitude group for organ index and body conformation measurements in accordance with NY/T 823-2020, Terminology and Statistical Methods for Poultry Production Performance [19]. Within each location, birds were first screened to exclude individuals showing overt disease, injury, abnormal body condition, or incomplete production records. Eligible birds were then randomly selected from the corresponding population using a random-number procedure based on individual cage or identification numbers. Selected chickens were humanely euthanized by electrical stunning in accordance with standard laboratory animal welfare guidelines to minimize distress. The heart, liver, spleen, lungs, and kidneys were immediately dissected. Adipose and connective tissues were removed, and each organ was weighed using an electronic balance with an accuracy of 0.01 g. Live body weight before slaughter was recorded for each bird. Organ index was calculated using the following formula:

(7) $$Organ\;index\;(\%)=\frac{Organ\;weight}{Live\;weight}\times 100$$

##### Body Conformation Traits

At 29 weeks of age, 12 healthy hens were randomly selected from the experimental population for comprehensive body conformation assessments, consistent with the protocols outlined in NY/T 823-2020, “Terminology and Statistical Methods for Poultry Production Performance”. Prior to measurement, each chicken was securely restrained. Measurements were performed using both a vernier caliper (accurate to 0.01 cm) and a flexible tape measure (accurate to 0.01 cm). All measurements were conducted by trained personnel using the same standardized procedure to reduce operator-related variation. To minimize bias in local morphological traits caused by differences in overall body size, body conformation measurements were standardized before statistical analysis. Because body oblique length was relatively stable between the two groups (*p* > 0.05), it was used as an internal reference for skeletal size. Relative indices were then calculated for the remaining body-size traits as follows: specific body-size measurement/body oblique length. The assessed parameters were defined as follows:

- Body oblique length: The linear distance extending from the anterior margin of the shoulder joint to the posterior margin of the ischial tuberosity.
- Chest depth: The vertical span from the apex of the dorsal spine to the inferior extremity of the sternum.
- Chest circumference: The perimeter encircling the broadest segment of the thoracic region.
- Chest width: The horizontal span between the bilateral shoulder joints.
- Keel length: The longitudinal extent from the anterior to the posterior terminus of the sternal keel process.
- Pelvic width: The horizontal distance separating the bilateral pubic bones.
- Shank length: The length from the proximal end of the tibia to the tarsal joint.
- Shank circumference: The circumference at the most robust point of the mid-tibial region.

### 2.4. Statistical Analysis

Statistical analyses were performed using SPSS 28.0. Prior to comparative analyses, all continuous data were assessed for normality using the Shapiro–Wilk test and for homogeneity of variance using Levene’s test. Variables that satisfied the assumptions of normality and homogeneity of variance were analyzed using the independent-samples Student’s *t*-test. For normally distributed variables exhibiting heterogeneity of variance, Welch’s *t*-test was applied. Variables that did not meet the normality assumption were evaluated using the nonparametric Mann–Whitney U test. Data are presented as the mean ± standard deviation (mean ± SD). Daily egg production rates were visualized using the matplotlib package in Python (version 3.11). Weekly production trends and physiological adaptation indicators were plotted using the ggplot2 package in R version 4.2.2.

## 3. Results

### 3.1. Laying Performance Differed Between Snowy White Chickens Reared at High and Low Altitudes

Table 1 summarizes the laying performance for Snowy White chickens reared under high- and low-altitude environments. Body weight at first laying did not differ significantly between the two groups (*p* > 0.05), whereas egg weight at first laying was significantly higher in the high-altitude group (*p* < 0.001). Furthermore, high-altitude rearing was associated with a delayed age at first laying compared to the low-altitude rearing.

**Figure 2:**
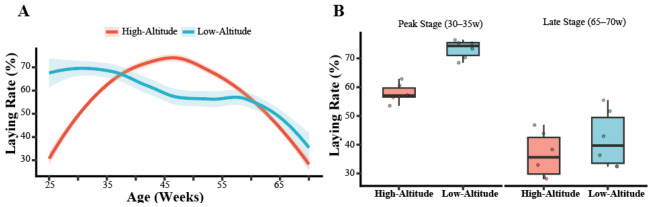
Weekly egg production dynamics of Snowy White chickens reared at high and low altitudes. The high- and low-altitude flocks consisted of 550 and 380 hens, respectively. (**A**) Weekly egg production rate calculated from flock-level production records. (**B**) Comparison of egg production rates during the peak laying period and late laying period. Circles represent individual data points, and the black horizontal line within each box represents the median.

Figure 2 shows the weekly laying-rate dynamics of Snowy White chickens reared under high- and low-altitude conditions. Based on flock-level production records, the low-altitude flock showed an earlier increase in laying rate and reached its apparent peak at approximately 30 weeks of age. In contrast, the high-altitude flock exhibited a delayed rise in egg production, with the apparent peak occurring at approximately 45 weeks of age. These descriptive longitudinal patterns suggest that high-altitude rearing was associated with delayed sexual maturity and postponed peak laying performance.

To further illustrate representative production stages, laying rates during weeks 30–35 and weeks 65–70 are shown in Figure 2B. Weeks 30–35 corresponded to the apparent peak-laying period of the low-altitude flock, during which the high-altitude flock was still in a delayed rising phase. Weeks 65–70 represented a later period when both flocks had entered a relatively stable laying stage. Because the weekly laying-rate data were derived from flock-level longitudinal records and were temporally correlated, these comparisons are interpreted as exploratory and descriptive rather than as independent repeated statistical tests across weeks.

### 3.2. Comparative Analysis of Hatching Performance in Snowy White Chickens at High and Low Altitudes

Table 2 summarizes the incubation performance of Snowy White chickens under high- and low-altitude conditions. The fertility rate did not differ significantly between the two environments (*p* > 0.05). However, the hatchability of set eggs and the hatchability of fertile eggs were both lower in the high-altitude group, although these differences did not reach statistical significance (0.05 < *p* < 0.1).

### 3.3. Organ Indices Differed Between Snowy White Chickens Reared at High- and Low- Altitudes

**Figure 3:**
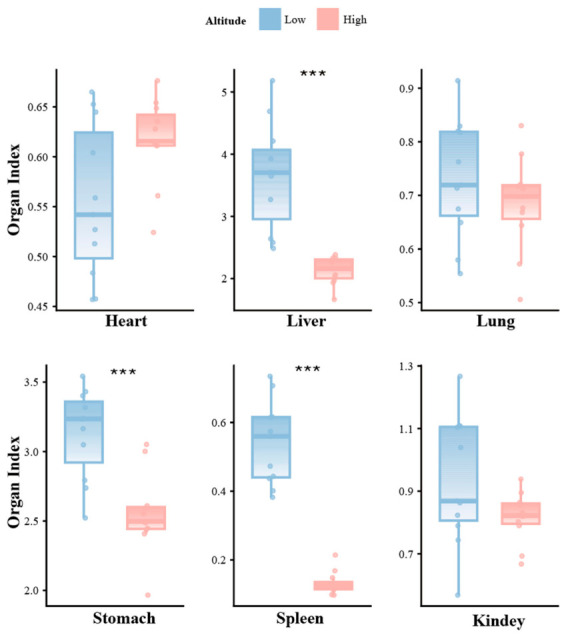
Comparison of organ indices in Snowy White chickens reared at high and low altitudes. Organ indices were measured in 12 clinically healthy hens randomly selected from each altitude group at 29 weeks of age (high altitude, *n* = 12 hens; low altitude, *n* = 12 hens). Each point represents an individual hen. *** *p* < 0.001.

Figure 3 shows organ indices for Snowy White chickens under different altitude environments. The heart index was higher in high-altitude birds than in low-altitude birds (*p* < 0.05). In contrast, liver, stomach, and spleen indices of chickens in the high-altitude group were significantly lower than those in the low-altitude group (*p* < 0.01). Lung and kidney indices did not differ significantly between the high- and low-altitude groups (*p* > 0.05).

### 3.4. Comparative Analysis of Body Conformation Traits in Snowy White Chickens at High and Low Altitudes

**Figure 4:**
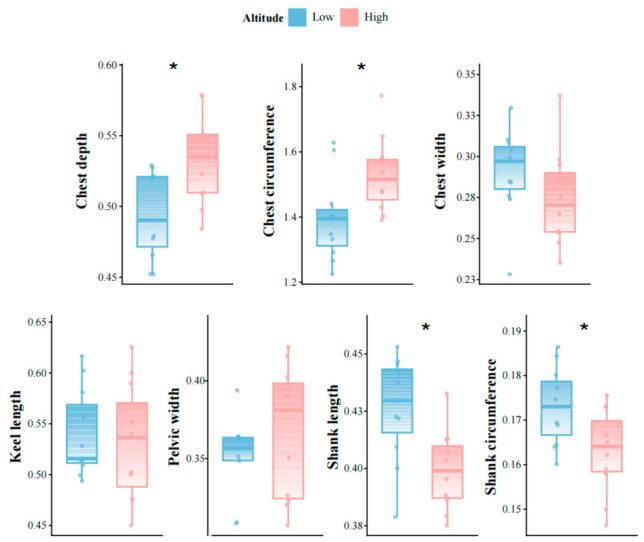
Comparison of body conformation traits in Snowy White chickens reared at high and low altitudes. Body conformation traits were measured in 12 clinically healthy hens randomly selected from each altitude group at 29 weeks of age (high altitude, *n* = 12 hens; low altitude, *n* = 12 hens). Each point represents an individual hen. * *p* < 0.05.

Figure 4 presents the comparative body measurement traits for Snowy White chickens under different altitude environments. Thoracic development parameters, including chest depth and chest circumference, were greater in the high-altitude group compared to the low-altitude group (*p* < 0.05). In contrast, leg development indicators, such as shank length and circumference, were significantly lower in the high-altitude birds than in low-altitude birds (*p* < 0.05). No significant differences were observed between the two groups in body oblique length, chest width, keel length, and pelvic width (*p* > 0.05).

## 4. Discussion

### 4.1. Impact of Altitude on the Production Performance of Snowy White Chickens

The Snowy White Chicken is currently the only light-type layer breed developed in Tibet and is considered adapted to high-altitude hypoxic environments. However, maintaining high production performance under chronic high-altitude hypoxia remains challenging [5]. Hypoxia is a defining feature of high-altitude regions and can affect metabolism and reproductive function in poultry. In the present study, age at first laying in the high-altitude group was delayed by 27 days, and peak egg production was postponed from approximately 30 to 45 weeks of age, indicating delayed reproductive maturation and peak production. In addition, hatchability of fertile eggs and hatchability of set eggs decreased by 10.50% and 9.97%, respectively. Such delays may increase early production inputs, including feed, vaccines, and labor, thereby reducing the production efficiency and profitability of layer production [20]. These findings are broadly consistent with previous reports showing impaired reproductive performance in chickens exposed to chronic hypoxia [21]. One possible explanation is that environmental stress associated with high-altitude rearing may influence the allocation of physiological resources related to growth and reproduction [5]. The lower laying rate observed in the high-altitude group further suggests that high-altitude conditions constrained reproductive output in Snowy White chickens [6]. In contrast, egg weight at first laying was higher in the high-altitude group. This may reflect altered maternal investment in individual eggs, which could increase embryonic reserves under hypoxic conditions and partly mitigate the adverse effects of the incubation environment [22,23]. While this may enhance the value of individual eggs, whether it can fully compensate for the challenges posed by hypoxic conditions remains to be determined.

The stable fertility rate observed in high-altitude birds suggests that fertilization capacity was relatively maintained under the conditions of this study. Nonetheless, avian embryonic development is highly sensitive to oxygen availability; hypoxia can suppress embryonic metabolism, disrupt organ differentiation, and reduce hatchability [8,24]. Research by Hassanzadeh et al. [25] showed that hypoxia induces oxidative stress, disrupts embryonic homeostasis, and increases embryonic mortality risk. Reduced oxygen availability in high-altitude regions is therefore an important factor influencing incubation duration and hatchability [26]. In the present study, Snowy White chickens maintained a fertile egg hatchability of 81.48% at 3650 m. Similarly, Zhang et al. reported a hatchability of 79.72% in Tibetan chickens at 2900 m, compared with only 31.69% in lowland dwarf chickens under the same conditions [6]. These findings support the reproductive resilience of chicken populations adapted to plateau environments under high-altitude conditions. However, these distinct profiles may not fully offset the adverse effects of hypoxia on late stage of embryonic development.

### 4.2. Physiological and Morphological Differences Observed Under High-Altitude Rearing Conditions

Hypoxia constitutes the principal environmental stressor at high altitudes, significantly constraining oxygen uptake, transport, and utilization [27]. In the present study, Snowy White chickens reared at high altitude exhibited a higher heart index, consistent with phenotypic patterns previously reported in other high-altitude animal studies [28]. Previous studies have reported cardiac structural and functional alterations in animals exposed to chronic hypoxic environments [29]. This may partly explain why some high-altitude poultry breeds retain a certain level of egg-laying capacity despite growth constraints [30]. By contrast, liver, stomach, and spleen indices were lower in the high-altitude group than in the low-altitude group. Under hypoxic conditions, metabolic resources may be preferentially allocated to the cardiopulmonary system at the expense of anabolic and digestive functions [31], while splenic adjustments may contribute to altered oxygen-carrying capacity [32]. These findings indicate that Snowy White chickens reared under the two environmental conditions exhibited distinct organ-development profiles, although the underlying physiological mechanisms remain unclear [1,33,34].

Alterations in body conformation further corroborate this phenotypic adaptation strategy. An enlarged thoracic cavity is hypothesized to facilitate increased respiratory volume and oxygen uptake efficiency, which is frequently observed as a morphological adaptation in high-altitude animals [35]. In the present study, the increased chest circumference observed in the high-altitude group may indicate increased thoracic capacity, which could provide anatomical support for cardiopulmonary adaptation [36,37]. In contrast, shank length and shank circumference were significantly diminished in the high-altitude group. Although reduced limb size may help limit body surface area, heat loss, and basal energy expenditure in cold or hypoxic environments [38], it may also affect mobility, feed intake, and nutrient utilization [39,40]. This limitation impedes the full realization of production performance under large-scale farming conditions in cold high-altitude regions, thereby diminishing the industrialization potential and economic benefits of Snowy White chickens in such areas.

This study provides a systematic assessment of phenotypic adaptation in Snowy White chickens reared at different altitudes; however, several limitations remain. First, although management conditions were standardized as much as possible, the study compared two field sites rather than a fully controlled experimental system. Consequently, environmental factors associated with altitude could not be entirely disentangled within the current analysis. Therefore, the observed phenotypic differences should be interpreted as a response to the high-altitude rearing environment rather than as effects of any single environmental factor. Second, this study focused primarily on phenotypic and physiological traits and did not examine the underlying molecular mechanisms. Future studies should integrate genomic and transcriptomic analyses to precisely decouple genetic and environmental factors, thereby elucidating the molecular mechanisms governing the high-altitude adaptability of Snowy White chickens.

## 5. Conclusions

This comparative study demonstrates that Snowy White chickens reared under high-altitude conditions showed delayed sexual maturity, postponed peak egg production, and lower egg production performance than those reared under low-altitude conditions. However, a distinct response profile was observed, as indicated by increased egg weight at the onset of laying. In addition, differences in heart and thoracic development, together with reduced digestive and immune organ indices, suggest distinct physiological characteristics under high-altitude rearing conditions. Given the observational nature of this two-site comparison, these phenotypic differences should be interpreted as a holistic response to the plateau environment rather than as direct consequences of hypoxia alone. These findings provide practical guidance for breeding and management strategies in high-altitude layer production systems, particularly by emphasizing reproductive efficiency, delayed production schedules, and physiological adaptation traits.

## Figures and Tables

**Table 1:** Laying performance of Snowy White chickens at high and low altitudes.

Indicators	High Altitude	Low Altitude	*p*-Value
Weight at first laying (kg)	1.29 ± 0.14	1.31 ± 0.15	0.360
Egg weight at first laying (g)	47.29 ± 3.09 ^a^	40.52 ± 3.26 ^b^	<0.001
Age at first laying (d)	203	176	

^a,b^ Significant differences are indicated by different superscript letters.

**Table 2:** Hatching performance of Snowy White Chickens at high and low altitudes.

Indicators	High Altitude	Low Altitude	*p*-Value
Number of Eggs Set for Incubation (eggs)	400	490	
Fertility Rate (%)	93.91 ± 0.09	94.03 ± 0.15	0.301
Hatchability of Fertile Eggs (%)	81.48 ± 3.59	91.98 ± 5.76	0.055
Hatchability of Set Eggs (%)	76.52 ± 3.45	86.49 ± 5.45	0.056
Healthy Chick Rate (%)	98.30 ± 1.21	99.18 ± 0.43	0.302

## Data Availability

The original contributions presented in this study are included in the article. Further inquiries can be directed to the corresponding authors.

## Supplementary Materials

The following supporting information can be downloaded at: https://www.mdpi.com/article/10.3390/life16060912/s1, Table S1: Stocking density at different brooding stages; Table S2: Temperature and relative humidity during the brooding period; Table S3: Composition and nutrient levels of basal diets; Table S4: Immunization schedule.
