# Supplementary material for: Comparative Analysis of Production Performance and Physiological Responses in Snowy White Chickens Reared at Different Altitudes
Source: Life (Basel). 2026 May 28;16(6):912. doi: 10.3390/life16060912 (PMC13302352; doi:10.3390/life16060912)
Supplement: Supplementary file 1 [file life-16-00912-s001.zip › life-4262270-supplementary.pdf]

Supplementary **Table S1.** Stocking density at different brooding stages.

Age (weeks)	Stocking density (chickens/m²)
1–2	60
3–4	40
5–6	30

Supplementary **Table S2.** Temperature and relative humidity during the brooding period.

Age	Temperature (°C)	Relative humidity (%)
1–3 d	34–35	56–70
4–7 d	32–33	56–70
8–14 d	31–34	53–70
3 weeks	29–31	50–70
4 weeks	27	50–70
5 weeks	25	50–70
6 weeks	23	50–70

Note: After 6 weeks of age, the ambient temperature was maintained above 13 °C in winter and below 35 °C in summer.

Supplementary Table S3. Composition and nutrient levels of basal diets.

Starter phase (1–6 weeks)

Ingredient (%)	Content	Nutrient level	Value
Corn	59.77	Metabolizable energy (MJ/kg)	12.10
Soybean meal	33.70	Crude protein (%)	19.50
Soybean oil	2.40	Calcium (%)	0.95
Limestone	1.30	Available phosphorus (%)	0.40
NaCl	0.25	Methionine (%)	0.43
Choline chloride	0.10		
Dicalcium phosphate	1.30		
Methionine	0.18		
Premix[1]	1.00		
Total	100		

Grower phase (7–20 weeks

Ingredient (%)	Content	Nutrient level	Value
Corn	63.00	Metabolizable energy (MJ/kg)	11.80
Soybean meal	23.00	Crude protein (%)	15.49
Soybean oil	3.00	Calcium (%)	0.81
Wheat bran	6.80	Available phosphorus (%)	0.67

NaCl	0.20	Lysine (%)	0.73
Dicalcium phosphate	3.00	Methionine plus cysteine (%)	0.64
Premix[2]	1.00		
Total	100		

Laying phase (≥20 weeks)

Ingredient (%)	Content	Nutrient level	Value
Corn	62.70	Metabolizable energy (MJ/kg)	11.09
Soybean meal	26.30	Calcium (%)	3.50
NaCl	0.30	Crude protein (%)	16.61
Limestone	8.50	Non-phytate phosphorus (%)	0.35
$Ca(HCO_3)_2$	1.00	Digestible lysine (%)	0.85
Methionine	0.10	Digestible methionine (%)	0.35
Choline chloride	0.10		
Premix[3]	1.00		
Total	100		

Premix composition (per kg of diet)

Premix [1]:

Vitamin premix1supplied (per kg of diet): Vitamin A, 9500 IU; Vitamin D3, 4560 IU; Vitamin E, 38 mg; Vitamin K3, 3.8 mg; Vitamin B1, 4 mg; Vitamin B2, 7.0 mg; Vitamin B6, 6.5 mg; Vitamin B12, 0.03 mg; Folic acid, 1.8 mg; Biotin, 0.32 mg; Nicotinamide, 50 mg; Cu,10 mg; Fe,65 mg ; Zn,95 mg; Mn,100 mg; I,1 mg; Se,0.3 mg.

Premix [2]:

Vitamin premix2supplied (per kg of diet): Vitamin A,12000 IU; Vitamin D3,3000 IU, Vitamin E,24 mg, vitamin K3,3 mg, Vitamin B1,12.4mg; Niacin,45 mg; D-pantothenic acid,12 mg; VitaminB6,3.6 mg; D-biotin,0.09 mg; folate 1.2 mg; VB12,15 μg; Fe,80 mg; Cu,8 mg; Mn, 90 mg; Zn, 85 mg; I, 0.8 mg; Se, 0.3 mg; 98% L-lysine, 204 mg; 50% cholinesterase, 2.6 g; phytase,0.1 g.

Premix [3]:

Vitamin premix3supplied (per kg of diet):Vitamin A, 10000 IU; Vitamin D3, 3000 IU, Vitamin E, 20 mg, Vitamin B12, 5 mg; Vitamin K, 3.2 mg; Folic acid, 1.5mg; Biotin,2 mg; Pyridoxine, 8 mg; Nicotinic acid, 32.5 mg; Choline, 500 mg; Calcium pantothenate, 40 mg; Riboflavin, 8 mg; Thiamine,1 mg; Zn, 80 mg; Fe, 80 mg; Mn, 70 mg; I, 1 mg; Cu, 8 mg; Se, 0.3 mg.

Supplementary Table S4. Immunization schedule.

Laying phase (≥20 weeks)

Age (d)	Vaccine	Dose	Age (d)
1	ND + IB (live)	1 dose/chicken	1
7–8	ND (ZM10) + IB (H120, QX)<br>ND–IB oil-emulsion	1–1.5 doses<br>0.35 mL	7–8

18	Avian influenza (H5 + H7)	0.35 mL	18
	Fowl pox	2 doses	
25	ND–IB (live)	2 doses	25
	ND–IB–adenovirus (oil)	0.5 mL	
35	Avian influenza (H5 + H7)	0.35 mL	35
45	Infectious coryza	0.5 mL	45
70	ND (LaSota) + IB (H120)	2.5 doses	70
	ND–IB oil vaccine	0.5 mL	
90	Coryza	0.5 mL	90
	AE + pox	1 dose	
110	ND–IB (freeze-dried)	3 doses	110
	ND–IB oil vaccine	0.7 mL	
	AI (H5 + H7)	0.5 mL	
200	ND–IB	3.5 doses	200
	ND–IB oil-emulsion	0.5 mL	
	AI (H5 + H7)	0.5 mL	

* ND= Newcastle disease; IB= Infectious bronchitis; AI= Avian influenza
